# Comparison of In Vivo and In Vitro Digestibility in Donkeys

**DOI:** 10.3390/ani10112100

**Published:** 2020-11-12

**Authors:** Sonia Tassone, Riccardo Fortina, Emanuela Valle, Laura Cavallarin, Federica Raspa, Silvia Boggero, Domenico Bergero, Mauro Giammarino, Manuela Renna

**Affiliations:** 1 Department of Agriculture, Forest, and Food Sciences, University of Turin, 10095 Grugliasco, TO, Italy; sonia.tassone@unito.it (S.T.); silvia.boggero@gmail.com (S.B.); 2 Department of Veterinary Sciences, University of Turin, 10095 Grugliasco, TO, Italy; emanuela.valle@unito.it (E.V.); federica.raspa@unito.it (F.R.); domenico.bergero@unito.it (D.B.); manuela.renna@unito.it (M.R.); 3 Institute of Sciences of Food Production, National Research Council, 10095 Grugliasco, TO, Italy; laura.cavallarin@ispa.cnr.it; 4 Department of Prevention, Asl TO3, Veterinary Service, Area Animal Sanity, Via Torino 62, 10045 Piossasco, TO, Italy; mgiammarino@aslto3.piemonte.it

**Keywords:** donkey, in vivo digestibility, in vitro digestibility, Daisy^II^ incubator, fecal inoculum

## Abstract

**Simple Summary:**

Traditional in vivo methods of determining digestibility of feeds are expensive and time-consuming, and very few data are available for donkeys. The aim of this study was to verify if the in vitro method developed for the Ankom Daisy^II^ Incubator could produce accurate estimates of the in vivo dry matter and neutral detergent fiber digestibility of diets fed to donkeys. Four donkeys and four diets were used in the trial, and the experiment was repeated four times. Buffered donkey feces were used as an inoculum source for the assessment of digestibility in vitro, with an incubation time of 60 h. The obtained results showed that the Ankom Daisy^II^ Incubator ranked the diets in the same order as the in vivo method. However, in vitro values were lower than those obtained in vivo. The regression analyses used to predict in vivo estimates from in vitro data gave poor results and low precision. In conclusion, further studies, using different sample size and digestion times in vitro, are needed to verify if accurate prediction of in vivo feed digestibility can be obtained using the Ankom Daisy^II^ Incubator and donkey feces as inoculum.

**Abstract:**

We compared in vivo and in vitro dry matter (DM) and neutral detergent fiber (NDF) digestibility in donkeys using feces as microbial inoculum. Four donkeys were used in a 4 × 4 Latin square design with a 2 × 2 factorial arrangement of treatments. The animals were fed two types of hay, with or without flaked barley. For the in vivo procedure, total feces were collected for 6 days from each donkey; digestibility was calculated as the difference between ingested and excreted DM and NDF. For the in vitro procedure, donkey feces were buffered and used as microbial inoculum in an Ankom Daisy^II^ Incubator; digestibility was estimated after 60 h of incubation. In vivo results showed that the addition of barley to hays did not change the digestibility values. In vivo estimates were higher than in vitro ones. The equations used to predict in vivo estimates from in vitro data were not reliable (R^2^ = 0.47 and 0.21; *P* = 0.003 and 0.078 for NDF and DM digestibility, respectively). Further studies need to evaluate different sample size and digestion times.

## 1. Introduction

To date, in the world there are 43–44 million donkeys used for work; pack transport; pulling carts; farm tillage; drawing water and milling; cosmetics or pharmaceutical industry; milk and meat; and—just in industrialized countries—for recreation, breeding, and companionship [1]. The donkey is a monogastric herbivore, evolved to have a steady flow of dietary fiber moving through the gut at all times [2]. In comparison with horses, donkeys have a higher efficiency in digesting fiber of poor nutritional quality [3]. Their large intestine leads to a higher digestibility of organic matter, gross energy and fiber fractions than in horses or ponies [4,5,6]. According to Edwards et al. [7,8], donkeys have a fecal microbiota concentration and a community composition that differ from those of other equine types, and this diversity plays an essential role in fiber digestion. Liu et al. [9] studied the microbiota at different sites of the gastrointestinal tract of donkeys, finding a higher microbial concentration in the hindgut than in the foregut.

Despite the increasing interest in donkeys, studies on feed digestibility in this species are very limited. Most of the available data resulted from in vivo feeding trials [4,10,11,12,13]. Liu et al. [14] studied the effect of dietary forage on digestibility using the mobile nylon bag technique. Kidane et al. [15] predicted diet digestibility via fecal-NIR, with few successes. Carretero-Roque et al. [11] and Wood [16] studied the in vitro digestibility of feedstuffs for donkeys using the neutral cellulase plus gamanase technique developed by Ankom Technology [17]. Tassone et al. [18] demonstrated that donkey’s digestibility can be predicted, with good repeatability and reproducibility, using the Daisy^II^ Incubator (Ankom Technology) and donkey feces as source of microbial inoculum. These authors also showed that the digestibility of different feeds for donkeys requires different incubation times. Leng et al. [19] proposed an in vitro model to study equine gut microbiota that represented an alternative to in vivo research. Despite these studies, a reliable tool for predicting feed digestibility in donkeys is not yet available.

The objective of this study was to compare the in vivo dry matter (DM) and neutral detergent fiber (NDF) digestibility of some diets commonly used in donkey nutrition with in vitro data obtained using the Daisy^II^ Incubator and donkey feces as a source of microbial inoculum. We hypothesized that the in vitro method developed for the Daisy^II^ Incubator produces accurate estimates of the in vivo DM and NDF digestibility of diets fed to donkeys.

## 2. Materials and Methods

The trial was carried out at the Veterinary Teaching Special Unit of the Department of Veterinary Sciences (DVS) of the University of Turin (Italy). The protocol was designed according to the guidelines of the current European Directive (2010/63/EU) on the care and protection of animals used for scientific purposes.

### 2.1. Animals and Diets

Four female Ragusano donkeys (A, B, C, D), aged from 12 to 16 months, were selected from a private farm (“Asi Lait”; San Benigno Canavese (TO), Italy) and brought to the experimental barn of the DVS 4 weeks before the beginning of the trial for adaptation. Upon arrival, the donkeys were individually weighed. The initial live weight was equal to 193 ± 24.3 kg. The donkeys were dosed with a broad-spectrum deworming product (“Eqvalan Duo”, Boehringer Ingelheim Animal Health Italia S.p.A., Noventana (PD), Italy) at the arrival and regularly checked for fecal parasites. They were housed in a stable in 3 m × 4 m single boxes, on wood shavings. The donkeys had free access to clean and fresh water. During the whole trial, the donkeys had access to a sandy paddock: they were let to exercise and socialize in pairs for 1 hour during the adaptation periods, while they were hand walked by an operator for 10 minutes during the collection periods. The operator took care of avoiding intake of sand by the donkeys during paddock turnout.

The animals were fed four diets (6 kg as fed/head × day) according to a 4 × 4 Latin square experimental design with a 2 × 2 factorial arrangement of treatment. The diets were: (i) 1st cut meadow hay (H1); (ii) 88% 1st cut meadow hay + 12% flaked barley (H1B); (iii) 2nd cut meadow hay (H2); and (iv) 88% 2nd cut meadow hay + 12% flaked barley (H2B). In a previous digestibility trial conducted with horses [20], cereals were given at a rate commonly used in equine rations (i.e., 30% oat and 70% hay). In the current trial, we decided to use a lower concentration of cereals since the starch limit for digestion is not known in donkeys. In addition, we wanted to set up a forage-based diet to avoid using a concentrate diet that could potentially be harmful for the animals and lead to a reduction in feeding consumption time. In H1B and H2B, hay and barley were given together. The diets were divided in three meals a day and the leftovers were collected and weighed daily to calculate the daily dry matter intake (DMI). Each of the four experimental periods (4 × 28 days) included: 14 days of adaptation to the new diet (days 1 to 14), 6 days of total feces collection for the in vivo digestibility assessment (days 15 to 20), 1 day of feces collection from each donkey for the in vitro digestibility assessment (day 21), and 7 days of wash out (days 22 to 28). The week before the beginning of the first collection period as well as during the wash out, each donkey was fed the hay used for the subsequent collection period.

Before every period of feces collection, representative samples of the experimental diets were collected, then ground using a cutting mill (MLI 204; Bühler AG, Uzwil, Switzerland) and analyzed for DM, ash and crude protein (CP) according to AOAC International [21]. Ether extract (EE) was analyzed according to AOAC International [22]. The detailed methods are reported in Fortina et al. [23]. The Ankom^200^ Fiber Analyzer (Ankom Technology, Macedon, NY, USA) was used to determine NDF, acid detergent fibre (ADF) and acid detergent lignin (ADL), following the procedures of Mertens [24] for NDF and Van Soest et al. [25] for ADF and ADL. Non-fibrous carbohydrates (NFC) were calculated with the following formula: NFC = 100-(NDF + ash + CP + EE).

### 2.2. In Vivo Digestibility

**Figure 1:**
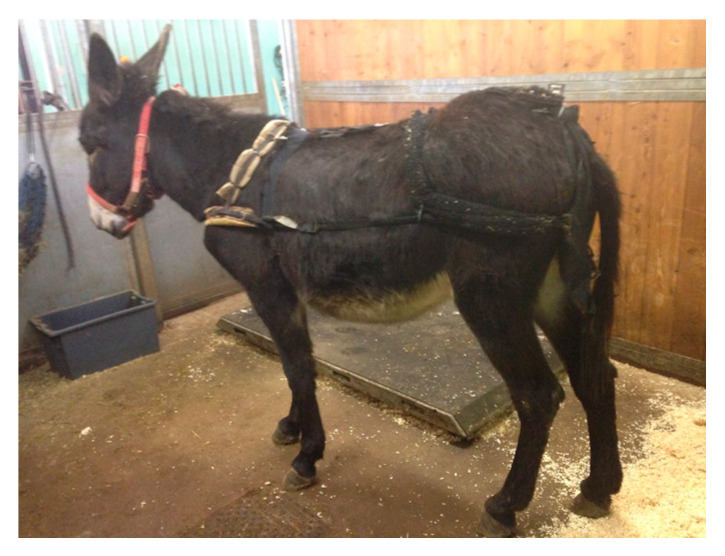
Donkey equipped with an adapted “Steed Apple Catcher”.

For the in vivo digestibility assessment, the donkeys were equipped with harnesses (“Steed Apple Catcher”; La Calèche, Bessemerstraat 370, 3620 Lanaken, Belgium) to allow total feces collection (Figure 1). The harnesses were adapted to the animals, according to their size, and fixed by an elastic rug girth fixed with a polo breastplate. The harnesses avoided the contamination of feces with urine. All the equipment was covered by a sheepskin cover tube as a pad protection.

For 6 consecutive days, the feces were removed from the harness five times a day (at 06:30, 09:00, 13:00, 17:00 and 22:00 h), then weighed and mixed. A daily 10% feces pool from each animal was dried at 50 °C; ground to pass 1 mm screen; and analyzed for DM, ash, CP, EE, NDF, ADF and ADL, following the same analytical procedures implemented for feed analyses [18].

The in vivo apparent DM digestibility (ADMD_vv_) was calculated as follows:

ADMD_vv_ (% DM) = ((DM_i_-DM_e_)/DM_i_) × 100 (1)

where:

DM_i_ (%) = ingested DM

DM_e_ (% DM) = excreted DM.

Similarly, the in vivo NDF digestibility (NDFD_vv_, % NDF) was calculated considering the ingested and excreted NDF, using the same calculation shown for ADMD_vv_. 

### 2.3. In Vitro Digestibility

In vitro digestibility was performed using a Daisy^II^ Incubator (Ankom Technology Corporation, Fairport, USA) and donkey feces as source of microbial inoculum. The Ankom Daisy^II^ Incubator (Ankom Technology Corporation, Fairport, NY, USA) is an alternative to traditional in vitro procedures. It is a thermostatically controlled chamber with four rotating digestion jars, where up to 92 samples (individually weighed in bags) could be analyzed simultaneously with inoculum and buffer solution. With this method, the material that disappears from the bag during the incubation is considered digestible. For the in vitro procedure, feces collection occurred on the 21st day of each experimental period, directly from the rectum of each donkey. While feces collection for the in vivo digestibility assessment occurred throughout the whole day, the feces used for the in vitro procedure were always collected at 09:00 h, with the aim of using the inoculum as soon as possible as to avoid microbial content decreases during time. Immediately after collection, the feces were sealed in individual airtight bags expelling air as much as possible to maintain anaerobic conditions [20,26], purged with CO_2_ and immediately transferred to the lab (approximately 3 min travel from the barn to the lab). During transport, the temperature of the feces was maintained at about 39 °C using a warm water-containing cooler.

Each jar of the incubator was assigned to a donkey, using its individual feces, and filled with buffer solution [27]. The buffer solution was obtained mixing two solutions in a 5:1 ratio (1500 mL of solution A: KH_2_PO_4_ 10 g/L, MgSO_4_^.^7H_2_O 0.5 g/L, NaCl 0.5 g/L, CaCl_2_^.^2H_2_O 0.1 g/L, CH₄N₂O 0.5 g/L; 300 mL of solution B: Na_2_CO_3_ 15.0 g/L, Na_2_S^.^9H_2_O 1.0 g/L). The inoculum was prepared mixing 200 g of feces of each donkey and 400 mL of buffer solution. The mixture was blended for 30 s (Osterizer Cyclo-Trol Eight; Oster, Moncalieri (TO), Italy), purged with CO_2_ for 15 s, and transferred to the respective digestion jar. The pH was adjusted to 7.0 using additional amounts of the buffer solution as suggested by Earing et al. [20]. Representative samples of the four diets were weighed (0.50 g) in Ankom F57 filter bags (Ankom Technology Corporation, Fairport, USA) and heat sealed. Each jar contained eight samples (two replicates/diet) and two blanks. All the diets were incubated together as no interaction effects were expected between grains and forage [28].

The incubation time was chosen considering previous studies that used the Ankom Daisy^II^ Incubator and equine feces as inoculum source [18,25,26]. In particular, in donkeys, Tassone et al. [18] showed that the in vitro DM and NDF digestibility generally increased from 30 to 72 h of incubation, but with remarkable variations among different feedstuffs. However, when evaluating the relationships existing between in vivo and in vitro (at 24-, 30-, 48- and 72-h incubation) digestibility estimates in horses, other authors obtained higher model fits when predicting in vivo estimates from in vitro data at 48 h rather than at 24 h [26] or at 72 h [20]. Considering that in donkeys the retention of feedstuffs is longer than in horses [4], and considering the lack of available comparisons between in vivo and in vitro estimates at incubation times included between 48 to 72 h in equines, we decided to use an incubation time of 60 h.

At the end of the digestion, the bags were removed from each jar, rinsed thoroughly with cold tap water and placed in a 50 °C forced-air oven to dry for 24 h. The bags were weighed and then analyzed for NDF with the Ankom^200^ Fiber Analyzer (Ankom Technology Corporation, Fairport, USA).

The in vitro apparent DM digestibility (ADMD_vt_) and NDF digestibility (NDFD_vt_) were calculated as follows:

ADMD_vt_ (% DM) = 100 × (DM_0h_ − DM_residue_)/DM_0h_ (2)

NDFD_vt_ (% NDF) = 100 × (NDF_0h_ − NDF_residue_)/NDF_0h_, (3)

where:

DM_0h_ (%) = dry matter ante incubation

DM_residue_ (% DM) = dry matter post incubation 

NDF_0h_ (% DM) = neutral detergent fibre ante incubation

NDF_residue_ (% NDF) = neutral detergent fibre post incubation.

### 2.4. Statistical Analysis

All data were statistically analyzed using SAS [29]. 

The proximate constituents of the diets were analyzed by one-way ANOVA, using the following model:

Y_ij_ = μ + α_i_ + ε_ij,_ (4)

where: Y_ij_ = observation; μ = overall mean; α_i_ = fixed effect of diet (H1, H1B, H2, H2B); ε_ij_ = residual error. The Tukey’s post hoc test was used for multiple comparisons.

Digestibility and DMI data were analyzed with the PROC GLM procedure considering diet (α) as a fixed factor, while donkey (β) and period (δ) were considered as random factors. The following model was used:

Y_ijkl_ = μ + α_i_ + β_j_ + δ_k_ + ε_ijkl_, (5)

where: Y_ijkl_ = observation; μ = overall mean; α_i_ = effect of diet (H1, H1B, H2, H2B); β_j_ = effect of donkey (A, B, C, D); δ_k_ = effect of period (1, 2, 3, 4); ε_ijkl_ = residual error. The *t* test was used for multiple comparisons.

For each diet, linear regression analysis (PROC REG procedure in SAS) was used to assess the relationships existing between in vivo and in vitro ADMD and NDFD.

Significance was set at *p* < 0.05.

## 3. Results

### 3.1. Diet Characteristics and Dry Matter Intake

The chemical composition of the experimental diets is reported in Table 1.

Hays (H1 and H2) showed significant differences in their chemical composition. In particular, H1 showed higher levels of NDF and ADF and NFC, and contemporarily lower levels of ash, CP and EE than H2. Comparable levels of DM and ADL were observed between hays. The same differences and similarities were observed when comparing diets H1B and H2B. Compared to hays, the addition of barley resulted in a significant increase of NFC, and a decrease of NDF, ADF and ADL. When barley was added to the 1st cut meadow hay (H1B), CP increased; on the contrary, the amount of CP decreased when barley was added to the 2nd cut meadow hay (H2B). The EE was not significantly affected by the inclusion of barley in the diets.

The characteristics of the hays influenced the average DMI of the donkeys (*p* < 0.001). The DMI values were higher with the 2nd cut meadow hay (H2 and H2B diets; 5.0 ± 0.58 kg/head × day and 5.0 ± 0.37 kg/head × day respectively) than with the 1st cut meadow hay (H1 and H1B diets; 4.5 ± 0.79 kg/head × day and 4.4 ± 0.91 kg/head × day, respectively).

### 3.2. Effect of Diet on Dry Matter and Neutral Detergent Fiber Digestibility

Table 2 shows the variation of ADMD_vv_, ADMD_vt_, NDFD_vv_, and NDFD_vt_ among the four experimental diets. The ADMD_vv_ was significantly higher in H2 and H2B when compared to H1, while H1B showed intermediate values. The ADMD_vt_ ranked the diets in the following order: H2B > H2 > H1B = H1. The H2 and H2B diets showed significantly higher NDFD_vv_ values than H1 and H1B diets. The NDFD_vt_ significantly differed among all the diets, with the highest value found for H2, followed by H2B, then by H1, and finally H1B.

### 3.3. Comparison between In Vivo and In Vitro Digestibility

Table 2 shows the comparison between in vivo and in vitro ADMD and NDFD for each experimental diet. In all cases, in vitro values were significantly lower than in vivo values.

**Figure 2:**
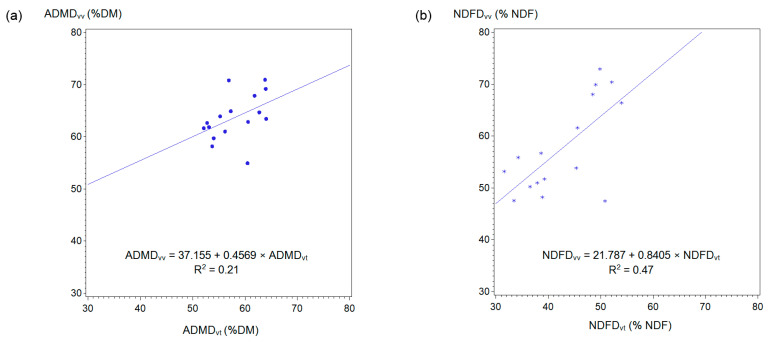
Relationship between 60-h in vitro digestibility using the Daisy^II^ Incubator and in vivo digestibility. (**a**) Dry matter digestibility. (**b**) Neutral detergent fiber digestibility. ADMDvv, apparent in vivo dry matter digestibility; ADMD_vt_, apparent in vitro dry matter digestibility; NDFD_vv_, in vivo neutral detergent fiber digestibility; NDFD_vt_, in vitro neutral detergent fiber digestibility. DM, dry matter; NDF, neutral detergent fiber.

The prediction equations obtained for the estimation of in vivo digestibility from in vitro values are the following ones (Figure 2):

ADMD_vv_ (% DM) = 37.155 + 0.4569 × ADMD_vt_ (% DM) (R^2^ = 0.21; SE = 0.240; *P* = 0.078) (6)

NDFD_vv_ (% NDF) = 21.787 + 0.8405 × NDFD_vt_ (% NDF) (R^2^ = 0.47; SE = 0.238; *P* = 0.003). (7)

The equation for NDFD was statistically significant and showed higher fitting (R^2^ = 0.47) than the equation for ADMD (R^2^ = 0.21). However, in both cases, the regression analyses gave poor results and the model fits were considered not reliable.

## 4. Discussions

### 4.1. Dry Matter Intake

Regardless of the type of diet, the DMI of the donkeys was higher than 2% body weight (BW). According to Raspa et al. [30], typical DMI in donkeys ranges from 1.3% BW in summer to 1.7% BW in winter. The DMI values observed in this trial were higher than those reported by Smith and Burden [3] (3 kg/head × day) and by Burden and Thiemann [31] (1.3 to 1.8% BW), but similar to those observed by Smith and Pearson [32], who reported values ranging between 1.75% and 2.25% BW. For lactating Amiata donkeys (BW of 309 kg), Gatta et al. [13] reported an average DMI of 8 kg/day for diets characterized by average NDF and ADF values equal to 49% DM and 31% DM, respectively. Agbagla-Dohnani et al. [33] measured an average DMI of rice and barley straws equal to 16.2 g/kg BW. Pearson et al. [12] observed that the DMI decreased with the lowering of feed quality (lucerne > temperate grass hay > straw), with values ranging between 21.4 and 10.4 g/kg BW. Differences in DMI are common in donkeys and are related to body weight, diet composition, and physiological and environmental conditions. In our study, the observed differences in DMI are related to the characteristics of the hays that influenced the average DMI of the donkeys. In fact, the DMI values were higher with the 2nd cut meadow hay than with the 1st cut meadow hay.

### 4.2. Effect of Diet on Dry Matter and Neutral Detergent Fiber Digestibility

The digestibility of feeds is generally reported to be higher in donkeys than other equines thanks to their capacity to digest low-quality feeds, to the high retention time of feeds in the gut [10] and, probably, to differences in the composition of the hindgut microbiota. Cudderford et al. [4] carried out a trial to determine the differences between Thoroughbreds, Highland ponies, Shetland ponies, and donkeys in their ability to digest diets containing different levels of fiber and protein (molassed alfalfa and oat straw at different proportions). The results showed that all animals digested the components of the high-fiber diets less well than those of the low-fiber diets; donkeys were more efficient in digesting the fiber and retained feed residues longer in the gastro-intestinal tract than the other equids. Pearson et al. [10] provided molassed dehydrated alfalfa or oat straw ad libitum or given at 70% ad libitum intake to ponies and donkeys. The results confirmed that ponies ingested more forages than donkeys per unit BW at both levels of feeding, but the apparent digestibility of dietary DM, energy and fiber fractions were always higher in donkeys. Izraely et al. [34] fed five donkeys with alfalfa and hay, and the NDFD_vv_ values ranged from 50.9 to 54.2% NDF, with no significant differences between the fiber digestibility of the two fodders. These authors concluded that donkeys are able to compensate for the adverse nutritional conditions like wild ruminants rather than the other domestic equines. Edwards et al. [7] observed that both fecal microbial concentrations and community composition of donkeys were generally most distinct from those of other equine types (horse, donkey, horse × donkey and zebra), even if a core fecal microbiota exists across all the equines. Most of the bacterial taxa of this core lack cultured representation; their availability is essential to gain fundamental knowledge of the microbial functions that underpin the equine hindgut ecosystem and that of the donkey in particular. In another trial, Edwards et al. [8] found that donkey fecal microbiota differed from that of both pony and pony × donkey, and these differences were related to a higher abundance and diversity of taxa with known (or speculated) roles in fiber degradation. The findings of these trials suggest that the specific composition of hindgut microbiota of donkeys plays an essential role in fiber digestion. 

In our study, NDFD_vv_ values of H1 and H1B diets (average: 51.8% NDF) were comparable to those obtained by Izraely et al. [34], while higher values were found for diets containing 2nd cut meadow hay (H2 and H2B; average: 63.8% NDF). In Mexican donkeys, Carretero-Roque et al. [11] found higher DM digestibility in vitro than in vivo for diets based on oat straw (average 53.4% vs 45.0%) and maize stover (average 58.3% vs 43.3%), both supplemented with 15% alfalfa hay. Pearson et al. [12] compared in vivo digestibility of forages of different quality (low: barley straw; medium: temperate meadow hay; high: lucerne) in different species, including donkeys, fed ad libitum or restricted to about 75% ad libitum intake. They found that DM digestibility decreased with the decrease of forage quality. Our results confirm such findings, as ADMD was significantly lower in the diets containing 1st cut meadow hay, which showed lower quality when compared to the 2nd cut meadow hay (Table 1). In donkeys, the apparent digestibility coefficients for DM ranged from 0.67 to 0.48. For NDF, the lowest and highest digestibility coefficients were found in lucerne (0.47) and temperate meadow hay (0.59), respectively. In the present trial, the average ADMD_vv_ value (63.7% DM) was similar to that obtained by Pearson et al. [12] for lucerne.

In our trial, the addition of 12% flaked barley (720 g/d) to hays did not significantly modify the ADMD_vv_ (Table 2). Similarly, Liu et al. [14] did not find any difference in the digestibility of diets characterized by different forage-to-concentrate ratios fed to donkeys. In contrast, other authors showed that the addition of a concentrate increased the digestibility of diets fed to horses up to approximately 10%. Palmgren Karlsson et al. [35] observed that the digestibility of DM, organic matter and energy increased in a curvilinear pattern with increasing inclusion levels of oat to grass-based diets fed to adult Standardbred geldings. However, the digestibility of fibrous components lowered at increasing inclusion levels of oats because of negative associative effects between grass hay and oats. Peiretti et al. [36] compared the in vivo digestibility of DM, organic matter, CP, NDF, and ADF of rations fed to horses based on hay and hay supplemented with flaked or crushed barley. They concluded that the addition of technological treated barley, and in particular flaked barley, increased total digestibility.

De Marco et al. [37] compared the in vivo and in vitro (pepsin-cellulase technique) apparent digestibility of broken rice added to a hay-based diet fed to adult geldings. The cereal showed high organic matter digestibility coefficients (92.6% in vivo and 87.1% in vitro) and its supplementation increased the digestibility of the diet. 

High amounts of grains or starch may lead to intestinal disorders and have negative impacts on digestibility of DM and dietary fiber in equids [38,39]. We did not find studies related to this topic on donkeys. However, the impact of high amounts and different barley forms on total tract apparent digestibility of dietary fiber in horses was assessed by Philippeau et al. [40]. In this trial, in the morning meal the animals were fed a concentrate (1.7% BW) made of 80% barley, distributed either as whole grain or as ground, pelleted or steam-flaked. Results showed that diets including treated barley forms led to a higher total tract apparent digestibility of NDF. Providing horses with high amounts of pelleted or flaked barley limited the negative impact of starch on fiber digestibility when animals were fed a high level of starch in the morning meal.

### 4.3. Comparison between In Vivo and In Vitro Digestibility

This is the first trial comparing in vivo and in vitro digestibility in donkeys using the Ankom Daisy^II^ Incubator and feces as a source of microbial inoculum. In 2005, Carretero-Roque et al. [11] also compared in vivo digestibility in donkeys with an in vitro method, but in that case the neutral cellulase plus gamanase technique [17] was used. Only two published studies estimated feed digestibility in equines using this closed system fermentation apparatus developed by Ankom Technology, and both of them were conducted on horses [20,26].

In our trial, in vivo values were higher than in vitro estimates. The in vitro method ranked the diets in the same order as the in vivo method for both ADMD and NDFD. Similarly, Carretero-Roque et al. [11] found the same trend between in vivo and in vitro digestibility estimates. Earing et al. [20] compared the in vivo and in vitro DMD and NDFD of four diets for horses at three incubation periods (30, 48 and 72 h) using the Daisy^II^ Incubator and horse feces as inoculum source. For three diets, the 72-h in vitro DMD and in vivo DMD values were not significantly different. The 30- and 48-h in vitro estimates were consistently lower than the in vivo estimates, but they ranked diets in the same order as the in vivo method. For NDFD, only the 72-h in vitro NDFD estimate of one diet was not significantly different when compared to the in vivo estimate. Earing et al. [20] concluded that the horse feces can be used in the Daisy^II^ Incubator to estimate in vivo DMD of horse feeds, and the 72-h incubation period provided digestibility estimates most similar to the in vivo data.

Comparing in vivo and in vitro digestibility estimates in donkeys, Carretero-Roque et al. [11] showed a significant (*p* < 0.05) regression equation with reliable model fit (R^2^ = 0.66). These authors concluded that in vitro analysis using the neutral cellulase plus gamanase technique could predict in vivo digestibility of donkeys fed low-quality forages. Conversely, in the current trial, the regression analysis showed that 60-h incubation length in a Daisy^II^ Incubator did not provide accurate estimation of in vivo DM and NDF digestibility (R^2^ = 0.21 and 0.47 for AMDM and NDFD, respectively). When compared to our results on donkeys, Earing et al. [20] showed in horses higher model fit for ADMD at all incubation times (R^2^ = 0.71, 0.70 and 0.63 at 30-, 48- and 72-h of incubation, respectively). On the contrary, for NDFD, the prediction equation obtained for donkeys in our trial showed less bad fit than those obtained by Earing et al. [20] for horses (R^2^ = 0.18, 0.06 and 0.06 at 30-, 48- and 72-h of incubation, respectively). 

Digestibility measured in vitro using the Ankom Daisy^II^ Incubator is influenced by different sources of variation, such as type of inoculum, time and temperature of its storage, type of bag, quantity of sample, time of digestion, physical conditions, and diet of the donor [41]. In our study, most of these conditions were fixed and under control. We can hypothesize that the limitations could be attributed to the weight of the sample and/or the time of digestion. Lattimer et al. [26] showed that in vivo digestibility on horses was predicted more accurately with an in vitro incubation of 48 h than 24 h (*p* < 0.05) and using 0.25 g instead of 0.50 g of sample. According to Cuddeford et al. [11], the estimated retention time (h) in donkeys fed with diets based on alfalfa or oat straw is equal to 76.7 and 53.8, respectively. An in vitro incubation time longer than 60 h may be required to obtain reliable prediction of in vivo digestibility data. In particular, further studies should be designed to determine the retention time of the feeds in donkeys, then using those times as incubation periods for in vitro digestibility trials. Another possibility could be that soluble substances were escaped from the F57 filter bags, influencing microbial population and consequently digestion, as found by Wilman and Adesogan [42].

## 5. Conclusions

In vivo DM and NDF digestibility values obtained through total feces collection were higher than the corresponding in vitro values obtained incubating donkey feces as inoculum source for 60 h in a Daisy^II^ Incubator. The in vitro method ranked the experimental diets in the same order as the in vivo method. However, the regression analysis did not show reliable prediction of in vivo estimates from in vitro data for both digestibility parameters. Further studies are needed to assess if the use of different sample size and/or digestion times with the Daisy^II^ Incubator would produce accurate estimates of in vivo DM and NDF digestibility when using donkey feces as inoculum source.

## Figures and Tables

**Table 1:** Chemical composition of the diets (g/100 g DM, unless otherwise stated; n = 4).

Item	H1	H1B	H2	H2B	SEM	*p*
DM (g/100 g as fed)	89.9	89.7	89.9	89.5	0.8	0.899
Ash	6.4 ^c^	6.1 ^c^	9.1 ^a^	8.0 ^b^	0.2	<0.001
CP	8.5 ^d^	9.7 ^c^	18.6 ^a^	17.5 ^b^	0.1	<0.001
EE	1.3 ^b^	1.5 ^b^	2.3 ^a^	2.1 ^a^	<0.1	<0.001
NDF	59.4 ^a^	54.2 ^c^	58.2 ^b^	49.8 ^d^	0.2	<0.001
ADF	36.6 ^a^	29.7 ^c^	33.3 ^b^	27.3 ^d^	<0.1	<0.001
ADL	4.4 ^a^	2.4 ^b^	4.2 ^a^	2.1 ^b^	0.2	<0.001
NFC	24.4 ^b^	28.5 ^a^	11.8 ^d^	22.6 ^c^	0.4	<0.001

Abbreviations: H1, 1st cut meadow hay; H1B, 88% 1st cut meadow hay + 12% flaked barley; H2, 2nd cut meadow hay; H2B, 88% 2nd cut meadow hay + 12% flaked barley; DM, dry matter; CP, crude protein; EE, ether extract; NDF, neutral detergent fiber; ADF, acid detergent fiber; ADL, acid detergent lignin; NFC, non-fibrous carbohydrates; SEM, standard error of the mean. Different superscripts (^a–d^) within a row indicate significant differences.

**Table 2:** Effect of diet on in vivo and in vitro dry matter and neutral detergent fiber digestibility. Comparison between in vivo and in vitro dry matter digestibility (ADMD_vv_ *vs* ADMD_vt_) and neutral detergent fiber digestibility (NDFD_vv_ *vs* NDFD_vt_) for each diet.

Diets	ADMD_vv_ (% DM)	ADMD_vt_ (% DM)	*t*	*p*	NDFD_vv_ (% NDF)	NDFD_vt_ (% NDF)	*t*	*p*
H1	60.4 ^b^	53.3 ^c^	6.34	<0.001	51.7 ^b^	38.3 ^c^	8.14	<0.001
H1B	63.1 ^a,b^	55.4 ^c^	5.47	<0.001	51.9 ^b^	34.3 ^d^	8.91	<0.001
H2	64.6 ^a^	60.5 ^b^	2.36	0.022	64.3 ^a^	51.7 ^a^	4.75	<0.001
H2B	66.6 ^a^	63.1 ^a^	2.19	0.033	63.3 ^a^	47.1 ^b^	6.21	<0.001
SEM	5.514	4.527			7.409	7.809		

Abbreviations: ADMD_vv_, apparent in vivo dry matter digestibility; ADMD_vt_, apparent in vitro dry matter digestibility; NDFD_vv_, in vivo neutral detergent fiber digestibility; NDFD_vt_, in vitro neutral detergent fiber digestibility; DM, dry matter; NDF, neutral detergent fiber; H1, 1st cut meadow hay; H1B, 88% 1st cut meadow hay + 12% flaked barley; H2, 2nd cut meadow hay; H2B, 88% 2nd cut meadow hay + 12% flaked barley; SEM, standard error of the mean. Different superscripts (^a–d^) within a column indicate significant differences among the diets (*p* < 0.05). Within each row and parameter (ADMD or NDFD), means with a *p* value lower than 0.05 are significantly different.
